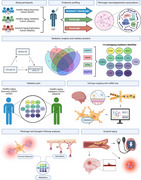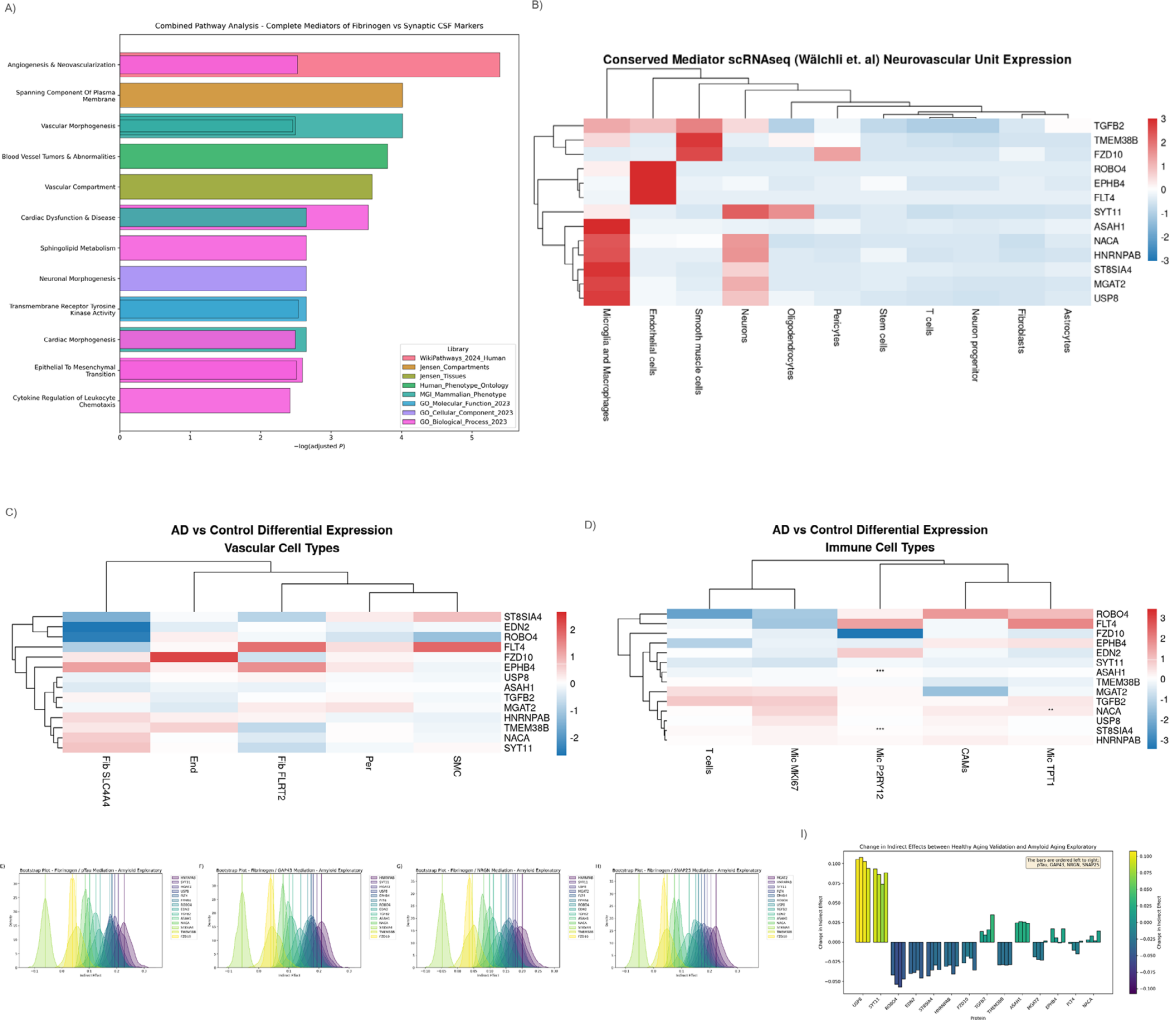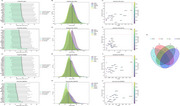# Vascular plasticity and microglial mediators link blood‐brain barrier dysfunction to synaptic decline in cognitively typical aging independent of amyloid

**DOI:** 10.1002/alz70856_107288

**Published:** 2026-01-10

**Authors:** Jonah Nadelmann Keller, Hannah Radabaugh, Nikolaos Karvelas, Nivetha Brathaban, Caleb H Radtke, Claudia E Kunney, Anna M Torten Rabinowitz, Harrison W Chan, Kaitlin B Casaletto, Henrik Zetterberg, Carlos Cruchaga, Adam Ferguson, Joel H Kramer, Katerina Akassoglou, Fanny M Elahi

**Affiliations:** ^1^ Icahn School of Medicine at Mount Sinai, New York, NY, USA; ^2^ Weill Institute for Neurosciences, University of California, San Francisco (UCSF), San Francisco, CA, USA; ^3^ Memory and Aging Center, Weill Institute for Neurosciences, University of California, San Francisco, San Francisco, CA, USA; ^4^ Memory and Aging Center, UCSF Weill Institute for Neurosciences, University of California, San Francisco, San Francisco, CA, USA; ^5^ UCSF Alzheimer's Disease Research Center, San Francisco, CA, USA; ^6^ Global Brain Health Institute, University of California San Francisco, San Francisco, CA, USA; ^7^ Institute of Neuroscience and Physiology, Sahlgrenska Academy at the University of Gothenburg, Göteborg, Sweden; ^8^ Dementia Research Centre, Department of Neurodegenerative Disease, UCL Queen Square Institute of Neurology, University College London, London, United Kingdom, London, United Kingdom; ^9^ Hong Kong Center for Neurodegenerative Diseases, Hong Kong, China; ^10^ Department of Neurology, Washington University School of Medicine, St. Louis, MO, USA; ^11^ NeuroGenomics and Informatics Center, Washington University School of Medicine, St. Louis, MO, USA; ^12^ Hope Center for Neurological Disorders, Washington University in St. Louis, St. Louis, MO, USA; ^13^ Department of Genetics, Washington University School of Medicine, St Louis, MO, USA; ^14^ The Charles F. and Joanne Knight Alzheimer Disease Research Center, St Louis, MO, USA; ^15^ Washington University School of Medicine, St. Louis, MO, USA; ^16^ Department of Neurology, Memory and Aging Center, University of California San Francisco, San Francisco, CA, USA; ^17^ Gladstone Institutes, UCSF, San Francisco, CA, USA

## Abstract

**Background:**

Cognitive decline frequently occurs during typical aging, yet the molecular mechanisms underlying this process remain poorly understood, especially in the absence of classical neuropathology such as amyloid. Vascular dysfunction and increased blood‐brain barrier (BBB) permeability have emerged as potential contributors. This study examined whether thromboinflammation, indicated by fibrinogen levels in cerebrospinal fluid (CSF), affects synaptic health and cognition in amyloid‐negative, typical aging.

**Method:**

We measured CSF fibrinogen and synaptic/neurodegenerative markers (pTau, GAP43, NRGN, and SNAP25) in cognitively normal older adults (*N* = 70). An unbiased proteomic screen (SomaScan 7k) was then performed to identify specific mediators linking fibrinogen to these markers, followed by validation in an independent cohort (*N* = 482). To assess potential interactions with amyloid pathology, we further examined these mediators in a separate group of amyloid‐positive older adults (*N* = 752).

**Result:**

Higher CSF fibrinogen was significantly associated with elevated pTau, GAP43, NRGN, and SNAP25, independent of amyloid status. Mediation analysis revealed 14 proteins that consistently linked fibrinogen to neurodegeneration, including factors regulating vascular plasticity and immune activation. Cell‐type mapping showed enrichment in endothelial cells, pericytes, and microglia. Results were validated in a large, independent typical aging cohort, confirming core pathways of fibrinogen‐mediated neurovascular dysfunction. In the amyloid‐positive cohort, vascular‐specific mediators were relatively reduced, indicating possibly altered molecular mechanisms in amyloid aging compared with typical aging.

**Conclusion:**

These findings highlight a thromboinflammatory pathway underlying synaptic decline in typically aging older adults, driven by fibrinogen at the neurovascular interface. Targeting BBB integrity and its vascular‐immune signaling may offer therapeutic opportunities to preserve cognition in aging, even in the absence of amyloid pathology.